# SS-31 peptide enables mitochondrial targeting drug delivery: a promising therapeutic alteration to prevent hair cell damage from aminoglycosides

**DOI:** 10.1080/10717544.2017.1402220

**Published:** 2017-12-07

**Authors:** Xiao Kuang, Shuang Zhou, Weiling Guo, Zhenjie Wang, Yanhui Sun, Hongzhuo Liu

**Affiliations:** School of Pharmacy, Shenyang Pharmaceutical University, Shenyang, P.R. China

**Keywords:** Mitochondrial targeting delivery, SS-31, aminoglycoside, hearing loss, geranylgeranylacetone, zebrafish lateral line

## Abstract

Aminoglycoside-induced hearing loss stems from damage or loss of mechanosensory hair cells in the inner ear. Intrinsic mitochondrial cell death pathway plays a key role in that cellular dysfunction for which no proven effective therapies against oto-toxicities exist. Therefore, the aim of the present study was to develop a new mitochondrial targeting drug delivery system (DDS) that provided improved protection from gentamicin. Particularly, SS-31 peptide-conjugated geranylgeranylacetone (GGA) loaded poly(lactic-co-glycolic acid) (PLGA) nanoparticles were constructed successfully via emulsion-solvent evaporation method. The zebrafish lateral line sensory system was used as an *in vivo* evaluating platform to investigate the protective efficiency against gentamicin. SS-31 modification significantly reduced the activity of mechanoelectrical transduction (MET) channel and gentamicin uptake in zebrafish lateral line hair cells. As expected, SS-31 conjugated nanoparticles showed mitochondrial specific accumulation in hair cells when compared with unconjugated formulations. Furthermore, intracellular SS-31 modified PLGA NPs slightly enhanced mitochondrial membrane potential (MMP, ΔΨ_m_) and then returned to a steady-state, indicating their effect on the respiratory chain complexes in mitochondria. GGA loaded SS-31 conjugated nanoparticles demonstrated the most favorable hair cells survivals against gentamicin when compared with unconjugated groups whereas blank formulations failed to exhibit potency, indicating that the efficiency was attributed to drug delivery of GGA. These results suggest that our constructed mitochondria-targeting PLGA based DDS have potential application in protecting hair cells from ototoxic agents.

## Introduction

Aminoglycosides are widely successful antibiotics in the clinical use due to their high efficacy against gram-negative bacterial infection. Despite their antimicrobial outcomes, all approved aminoglycosides by FDA are associated with adverse effects, which prominently target the kidneys and auditory organs. While nephrotoxicity is reversible and could be clinically controlled by hydration therapy, ototoxicity is permanent as damaged mechano-sensory hair cells (accounting for hearing and balance) cannot regenerate in humans and other mammals (Guthrie, 2008; Xie et al., 2011).

Among aminoglycoside class, gentamicin is one of the most frequently prescribed antibiotics since it was proved most potent in the treatment of several types of bacterial infections, involved in bone infections (Fleiter et al., 2014), endocarditis (Bassetti et al., 2013), and life-threatening sepsis especially in newborns or immunocompromised persons (Thomson et al., 2003). It is therefore on the World Health Organization’s list of essential medicines. There is currently no approved drug or treatment to prevent gentamicin induced hearing loss.

From *in vitro* and *in vivo* studies, it is known that gentamicin damages hair cells in the cochlea, resulting in hearing impairment or even deafness (Huth et al., 2011). Therefore, developing strategies to protect hair cells is a primary and urgent goal for the prevention of gentamicin induced ototoxicity. An exact mechanism to elucidate gentamicin induced ototoxicity remains extensive debate, however, it has been suggested that this cellular dysfunction occurs through intrinsic mitochondrial cell death pathway (Dehne et al., 2002; Owens et al., 2007). Although multiple death and protective factors including reactive oxygen species (ROS), JNK signaling and heat shock proteins are involved (Yu et al., 2009; Wong & Ryan, 2015; Kingwell, 2016), the therapies aimed at governing homeostasis within mitochondria may be more effective at stemming aminoglycoside induced hearing loss than other general approaches. Several laboratories, including ours, have noted that mitochondrial targeted therapies are more effective at mitigating aminoglycoside toxicity (Ojano-Dirain & Antonelli, 2012; Ojano-Dirain et al., 2014; Esterberg et al., 2016). Recently, we demonstrated that triphenylphosphonium (TPP^+^) surface engineering nanoparticles can efficiently uptake into mitochondria, which afford more potent against gentamicin induced hair cells damage than general nanoparticles (Wang et al., 2017). However, TPP^+^ modified nanoparticles were toxic to hair cells at increased concentrations, thus compromising their bio-application in inner ear drug delivery.

Herein, we described a new development of mitochondrial targeting nanoparticles against gentamicin induced hearing loss that potentially locally deliver a heat shock protein inducer, geranylgeranylacetone (GGA) to protect hair cells. The active mitochondrial targeting ability of these nanoparticles was conferred by a unique mitochondrial homing peptide (d-Arg-2′,6′-dimethylTyr-Lys-Phe, also named Szeto-Schiller-31, SS-31) that is conjugated to their surface. SS-31 represents the first of a class of new chemical entities that selectively target mitochondrial cardiolipin and scavenge mitochondrial ROS (Szeto, 2014). It has been noted that SS-31 provides therapeutic benefits in mitochondrial disease, including cardiovascular, renal, ophthalmic, metabolic and skeletal muscle disorders (Szeto & Birk, 2014). To accelerate the clinical translation of nanomedicine, an FDA approved biomaterial, poly(lactide-co-glycolides) (PLGA) based formulation was used in the present study due to its superior biodegradable and biocompatible characters. Our data indicated that SS-31 peptide functionalization greatly improved protective efficacy of GGA encapsulated nanoparticles against gentamicin induced hair cell damage in zebrafish model. Interestingly, we found that SS-31 modified PLGA NPs inhibited gentamicin uptake in hair cells via mechanoelectrical transduction (MET). Intracellular SS-31 modified PLGA NPs slightly enhanced mitochondrial membrane potential (MMP, ΔΨ_m_) and then returned to a steady-state, indicating their effect to the respiratory chain complexes in mitochondria.

## Experimental

### Materials

SS-31 peptide with a cysteine on the N-terminal was purchased from GL Biochem Ltd. (Shanghai, China). Poly(lactic-co-glycolic acid) (PLGA-COOH, 50:50, M.W. 13,000) and methoxy terminated PLGA-poly(ethylene glycol) (PLGA-mPEG_2000_, 50:50, M.W. 13,000) were purchased from Daigang Biotechnology Co, Ltd. (Jinan, China). PLGA-PEG conjugated rhodamine by amide linkage (PLGA_13000_-PEG_3400_-rhodamine) was purchased from Shanghai Ponsure Biotech, Inc. (Shanghai, China). Amino-poly(ethylene glycol)-maleimidyl (NH_2_-PEG_3400_-Mal) was purchased from JenKem Co., Ltd. (Beijing, China). Geranylgeranylacetone was purchased from Meilunbio (Dalian, China). Gentamicin sulfate (GT) was bought from DongKangYuan Science and Technology Ltd. (Wuhan, China). N-hydroxysuccinimide (NHS), dicyclohexylcarbodiimide (DCC), and N, N-Diisopropylethylamine (DIPEA) were obtained from J&K Scientific (Beijing, China). Sodium cholate, 2-[4-(dimethylamino)-styryl]-1-ethylpyridinium iodide (DASPEI), and MS-222 were obtained from Sigma-Aldrich (St. Louis, MO). Texas Red (TR) succinimidyl ester and FM1-43FX were both purchased from Molecular Probes, Inc. (Invitrogen, Eugene, OR). 3, 3-Diethyloxacarbocyanine iodide (DiOC_2_(3)) was bought from Tokyo Chemical Industry Development Co., Ltd. (Tokyo, Japan). MitoTracker^®^ Green FM was obtained from YEASEN Biotechnology Co., Ltd. (Shanghai, China).

### Animals

Zebrafish embryos were obtained by paired matings of wild type adult fish (China Zebrafish Resource Center, Wuhan, China). Embryos were raised at 28.5 °C in embryo medium (EM) consisting of 5 mM of NaCl, 0.17 mM of KCl, 0.33 mM of CaCl_2_, and 0.33 mM of MgSO_4_ at a density of 50 animals per 100-mm^2^ Petri Dish. All zebrafish protocols were approved by the Shenyang Pharmaceutical University Institutional Animal Care and Use Committee. All methods were performed in accordance with the guidelines of the Animal Care Ethics Committee of Shenyang Pharmaceutical University Medical Center and National Institutes of Health (NIH) guidelines.

### Synthesis of Mal-PEG-PLGA

Maleimide functionalized Mal-PEG-PLGA was prepared by linking Mal-PEG-NH_2_ to PLGA-COOH through a carbodiimide-mediated coupling reaction according to previous reports (Luo et al., 2010). Briefly, PLGA–COOH (1 g) was added 4 mL dichloromethane in the presence of NHS (1:8 PLGA:NHS molar ratio) and DCC (1:8 PLGA:DCC molar ratio) to form an ester. The mixture was allowed to react at RT for 4 h. The resulting PLGA-NHS was then precipitated with ice-cold ethyl ether (5 mL), and repeatedly washed in an ice-cold mixture of ethyl ether and methanol to remove residual NHS and DCC. To the activated polymer was added maleimide–PEG–NH_2_ (1:1.3 PLGA:PEG molar ratio) in 4 mL dichloromethane. The resulting mixture was allowed to react under gentle stirring overnight in the presence of DIPEA (28 mg, 0.22 mmol). The polymer was washed with ice-cold methanol to remove unreacted PEG. The Mal–PEG–PLGA product was recovered using cold ethyl ether, vacuum dried for 2 h, and stored at −20 °C until use. The obtained modified polymer was identified and characterized using nuclear magnetic resonance spectroscopy (400 MHz ^1^H NMR, Bruker Corporation, Fällanden, Switzerland, Fig. S1).

### Preparation and characterization of nanoparticles

Nanoparticles made of a blend of mPEG–PLGA and maleimide–PEG–PLGA were prepared through the classic emulsion-solvent evaporation technique. In brief, a certain amount of GGA together with mPEG–PLGA or maleimide–PEG–PLGA (1/1 or 1/0, w/w, total 30 mg) was dissolved in dissolved in dichloromethane, and the oil phase was poured into 5 mL of 1.0% sodium cholate solution under probe sonication for 2 min. The emulsion was then added to another 35 mL of 0.5% sodium cholate under stirring to evaporate the organic solvent. NPs were then filtered through 1 μm membrane filters, collected by centrifugation (13,000×*g*, 30 min) at 4 °C, and thoroughly washed with water.

For the preparation of SS31-PEG-PLGA NPs, SS-31 with sulfhydryl group was conjugated to the maleimide function located at the distal end of PEG surrounding the NPs surface. SS-31-Cys was mixed with maleimide–PEG–PLGA at peptide:maleimide molar ratio of 1.3:1 in PBS 7.4. The mixture was allowed to react in a shaking bath with a low speed for 1 h. NPs were concentrated by centrifuging (13,000×*g*, 30 min) at 4 °C. In order to examine the conjugation efficiency of SS-31 to maleimide on the nanoparticles, Ellman’s reagent (5, 5′-dithiobis-(2-nitrobenzoic acid)) was used to estimate free sulfhydryl groups in supernatant as a measure of degree of thiolation, in which reagent reacts with free sulfhydryl groups to produce a mixed disulfide and 2-nitro-5-thiobenzoic acid, a yellow colored compound with absorbance at 412 nm (Mantovani et al., 2005). To confirm the conjugation of SS-31, the resident content of SS-31-Cys in supernatant after reaction was quantified by HPLC (experimental detail in supporting information). Besides, lyophilized SS31-PEG-PLGA NPs was also performed to conduct X-ray photoelectric spectroscopy (XPS) analysis (ESCALAB 250Xi spectrometer, Thermo Fisher, Waltham, MA) with Al Kα source (1486.6 eV) at 14.0 kV.

Particle size and *ζ*-potential of prepared NPs were measure by a dynamic light scattering detector (Zetasizer, Nano-ZS, Malvern, Worcestershire, UK). The morphology of NPs was detected by transmission electron microscopy (TEM, Tecnai G2 F20 S-Twin, FEI, Hillsboro, OR). The encapsulation efficiency and the loading efficiency of GGA were analyzed as previously described method (Wang et al., 2017). In brief, a known amount of GGA-loaded nanoparticles was dissolved in acetonitrile for HPLC measurement (Hitachi Limited, Tokyo, Japan). The analysis of GGA was performed on an ODS column (Inertsil ODS-SP C18 column,150 × 4.6 mm, 5.0 μm particle size, GL Sciences Inc., Tokyo, Japan) at 30 °C. The mobile phase was 93% acetonitrile and 7% purified water. Flow rate was 1.0 mL/min and the detection wavelength was set at 205 nm.

### *In vitro* release

Drug release was conducted in a transwell plate (Dinh et al., 2016). The NPs suspension (300 μL) was allowed to add into Corning incorporated transwell inserts (12-mm diameter, polycarbonate membrane, 0.4 μm pore size) that had previously been inserted into a 12-well plates. The inserts were incubated at 37 °C and 1 mL HEPES (pH 7.4) was added into the 12-well plate as the medium. The entire volume of release media was collected at predetermined intervals and replaced with another 1 mL of fresh release media. The GGA concentration in the collected release medium was measured by HPLC. All of the above operations were repeated in triplicate.

### Gentamicin uptake assay

To evaluate the impact of NPs on gentamicin uptake by hair cells, the 5 dpf (days post fertilization) zebrafish larvae were incubated for 1 h in diluted NPs, followed by a 10 min co-incubation with 100 μM gentamicin tagged with fluorophore Texas red (GTTR, fluorescently-labeled experimental detail see supporting information) (Steyger et al., 2003; Wang & Steyger, 2009). Excess fluorophore was removed with 2–3 rinses in fresh EM and the larvae were observed under a confocal laser scanning fluorescent microscopy (Leica, Heidelberg, Germany). Neuromast GTTR intensity was assessed qualitatively.

To assess the MET channel integrity, 5 dpf larvae were exposed to diluted NPs for 1 h and then 1 μM of FM1-43FX was added for the treatment for 45 s (Hailey et al., 2017). Followed by 3× rinses with fresh EM, neuromasts were then observed using Leica epifluorescent microscope at 3 min after addition of FM1-43 to assess uptake.

### Mitochondrial co-localization assay

The mitochondrial location of SS31-PEG-PLGA NPs was investigated using epifluorescent microscope. Briefly, the zebrafish larvae (5 dpf) were pretreated with fluorescently-labeled formulations (1% of mPEG-PLGA was substituted with PLGA-PEG-rhodamine) for 4 h and hair cell mitochondria were labeled by 100 nM of Mitotracker Green for 30 min. Then the fish were anesthetic and immobilized for observation (Owens et al., 2007; Hailey et al., 2017). Unconjugated nanoparticles were used as control.

### Mitochondrial membrane potential (ΔΨ_m_)

Mitochondrial activity is tightly regulated with ΔΨ_m_ under physiological conditions. We measured ΔΨ_m_ in lateral line hair cells using the potentiometric mitochondrial dye DiOC_2_(3). Briefly, zebrafish larvae at 6 dpf were immersed EM containing diluted NPs (1 h or 2 h) or 100 μM oligomycin (positive control) for 10 min or 1 μM FCCP (negative control) for 5 min. Before evaluation, larvae were immersed in EM containing 0.1 μM DiOC_2_(3) for 30 min. After the incubation, *in vivo* imaging of zebrafish was performed using a fluorescent microscope. The area of fluorescent signal of DiOC_2_(3) at the neuromasts was quantified (Sasagawa et al., 2016).

### Gentamicin induced hair cells damage model establishment

To establish dose-response hair cells damage, 5 dpf larvae were incubated with ototoxic agent for 1 h (termed ‘acute exposure’) or 6 h (termed ‘continuous exposure’) in a range of concentrations of gentamicin (Owens et al., 2009). The exposed larvae were then rinsed 4× with fresh EM. The treated larvae in the acute exposure were allowed to recover for one hour prior to hair cell assessment. On the contrary, the larvae in the continuous exposure were examined immediately after the exposure period.

### Pharmacological treatments

Response of mechanosensory hair cells of the zebrafish lateral line to the NPs was evaluated against gentamicin induced hair cell impairment. 5 dpf larvae were randomly classified into two sets, which were subjected to acute or chronic exposure (Owens et al., 2009; Kruger et al., 2016). For acute exposure, larvae were treated by 200 μM of gentamicin for 1 h; while in the chronic exposure, larvae were treated with 2 μM of gentamicin for 6 h. The concentration of gentamicin was chosen in each set reliably induced approximately 40–60% of hair cells damage. The larvae in each set were then randomly divided into seven groups: control (EM-treated group), Gen group (without oto-protective treatment), blank mPEG-PLGA NPs and SS31-PEG-PLGA NPs group, mPEG-PLGA NPs loaded with GGA group, SS31-PEG-PLGA NPs loaded with GGA group and free GGA group. Each group was cultured in 24 well tissue culture plates. For experiments with protective agents, larvae were pretreated for 1 h in EM medium supplemented with the formulations with 27 μM of GGA. Gentamicin was then added and larvae were treated acutely or chronically with the presence of the formulation. After the toxin exposure, the larvae were rinsed rapidly with fresh EM four times, then held in the final wash for another 1 h to recovery (for acute exposure paradigm only). Hair cell survival was assessed with DASPEI staining or direct hair cell counts by immunocytochemistry.

### Hair cells assessment

For rapid *in vivo* assessment, the hair cells of the zebrafish lateral line were labeled with the mitochondrial dye DASPEI. The post-treated larvae were incubated in the EM containing 0.005% DASPEI for 20 min prior to anesthetization with 0.02% of MS-222 (Harris et al., 2003). Then the larvae were rinsed 3× with fresh EM. DASPEI labeling was evaluated on a Leica epifluorescent microscope (Chroma Technologies, Brattleboro, VT) for 10 neuromasts (SO1, SO2, SO3, P1-4, MI1, O1, and O2) (Harris et al., 2003). Each neuromast was assigned a score of 0 (no/little staining), 1 (reduced staining), or 2 (wildtype-like staining) for a composite score of 0–20 (Owens et al., 2009). DASPEI scores were averaged for each group and normalized as a percentage of mock-treated controls.

Since the DASPEI assessment is semi-quantitative, the immunocytochemistry was used to validate the protection of NPs from gentamicin induced hair cell damage. Briefly, the post-treated larvae were euthanized with an overdose of MS-222 and fixed with 4% paraformaldehyde overnight at 4 °C. Then the larvae were rinsed in PBS and incubated in blocking solution (1% Triton-X, 5% normal goat serum (NGS) in PBS) for 1–2 h at RT. To label the hair cells, the larvae were incubated overnight in anti-par at 4 °C parvalbumin primary antibody (monoclonal, 1:500 in 1% Triton-X, 1% NGS, in PBS, GenTex, San Antonio, TX), then rinsed in 1% Triton-X in PBS (PBS-T) and transferred to Alexa 488 goat anti-rabbit fluorescent secondary antibody solution (1:500 in 1% Triton-X, 1% NGS, in PBS, Invitrogen, Eugene, OR) for a 2–4 h incubation at room temperature. Following 2–3 additional rinses in PBS-T, the larvae were stored in 1:1 PBS/glycerol at 4 °C prior to assessment on a Leica epifluorescent microscope. The hair cell number was quantified in five neuromasts (O1, O2, MI1, M2, and OP1) per larvae, summed to calculate one value per animal, and averaged for each group. Results were compared as the mean hair cell survival as a percentage of the group treated only in EM (Thomas et al., 2015).

### Statistical analysis

We used Origin 8.0 (OriginLab Inc., Northampton, MA) for the statistical analysis. All results were represented as mean ± standard deviation (mean ± SD). Hair cells counts were evaluated by *t*-test and one-way analysis of variance was used for multiple comparisons. Significance was accepted for *p* values <.05.

## Results

### Characterization of NPs

In the present study, we fabricated PLGA-PEG-NPs for SS-31 modification by mixed with maleimide-terminated PLGA_13K_-PEG_3.4K_ and methoxy PLGA_13K_-PEG_2K_ at a 1:1 ratio to form NPs via emulsion-solvent evaporation method. The SS-31 modified PLGA-PEG-NPs were then prepared by a conjugation of thiolated SS-31 and maleimide functional PLGA-PEG-NPs. The conjugation rate of SS-31 was monitored by using Ellman’s test and 84% of maleimide was conversed into thioether. The amount of SS-31 conjugated to NPs was also calculated by HPLC analysis after treatment with dithiothreitol, a disulfide-reducing reagent. The conjugation efficiency of SS-31 was thus estimated to be approximately 81.2%. High-resolution XPS was also carried out to further confirm the conjugation of SS-31 on the surface of NPs. As shown in Fig. S2, the percent of sulfur, which can be only related with thiolated SS-31, onto the surface of NPs increased (0.26 vs 0.16, Table S1). Furthermore, the oxygen content onto the surface of PLGA nanoparticles increased, indicating a preferential surface localization of SS-31 (Alonso-Sande et al., 2013).

**Figure 1.**
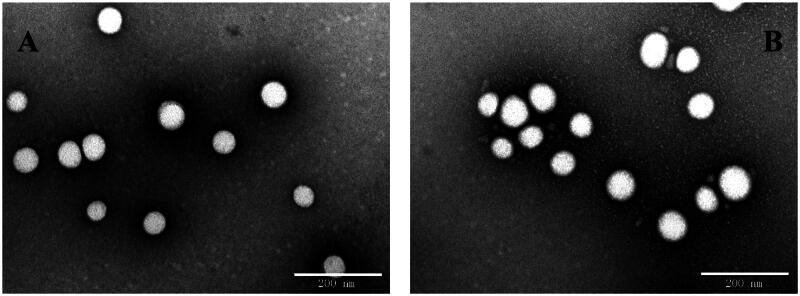
Representative TEM images for GGA-loaded NPs (A) and GGA-loaded SS31-NPs (B). Scale bar: 200 nm.

The physicochemical properties and drug loading parameters of GGA loaded PLGA NPs were determined and the results are shown in Table 1. The particle sizes of PLGA-PEG/GGA and SS31-PEG-PLGA/GGA was 105.8 ± 2.8 nm and 115.3 ± 0.8 nm respectively with a narrow distribution (PDI: 0.164 vs 0.150) as determined by DLS, which was further confirmed by TEM. Figure 1 shows that the NPs were well dispersed in water and exhibited spherical morphologies. After SS-31 modification, the mean size of NPs slightly increased (∼10 nm) and zeta potential decreased. The EE% of GGA loaded NPs was approximately 70% and the DL rate was 3.6–3.9% without any significant differences between them, suggesting that the drug loading ability remained after introduction of SS-31.

**Table 1.** Characterization of unmodified and SS-31 modified nanoparticles.

Sample	Size (nm)	PDI	Zeta (mV)	EE (%)^a^	DLC (%)^b^
Blank NPs	96.59 ± 1.537	0.159 ± 0.035	−12.6 ± 0.404	–	–
GGA loaded NPs	105.8 ± 2.822	0.164 ± 0.021	−16.1 ± 0.862	70.78 ± 9.581	3.617 ± 0.6155
SS31-PEG-PLGA NPs	103.1 ± 0.9899	0.133 ± 0.003	−5.93 ± 0.458	–	–
GGA loaded SS31-PEG-PLGA NPs	115.3 ± 0.7974	0.150 ± 0.024	−6.50 ± 0.308	71.42 ± 3.982	3.951 ± 0.4952

^a^ EE% is encapsulation efficiency.

^b^ DLC% is drug loading capacity.

### *In vitro* release

**Figure 2.**
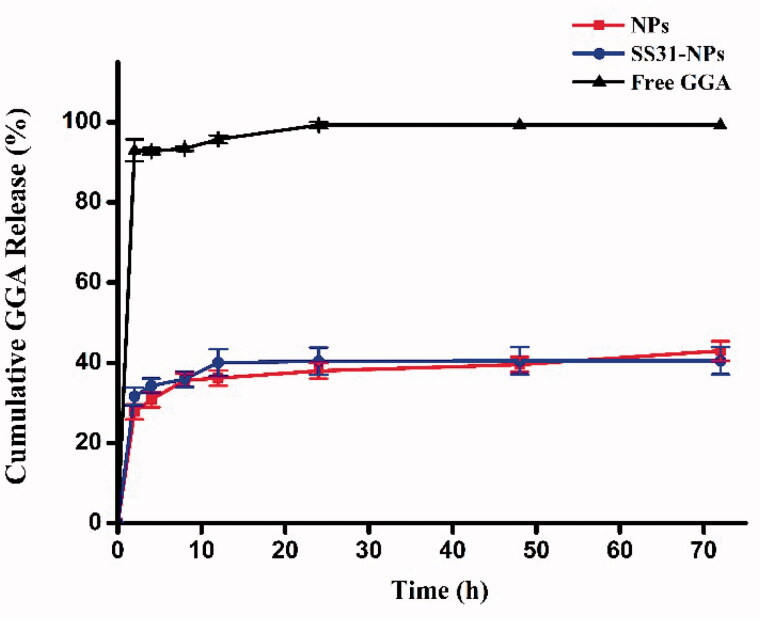
Cumulative release of GGA from nanoparticles in 20 mM HEPES (pH 7.4) with free drug as control.

Since GGA was relatively stable in HEPES, the release profiles of formulations was evaluated in HEPES (pH 7.4). As shown in Figure 2, we observed an initial burst release of 20% in the first 1 h for GGA loaded PLGA NPs, followed by a sustained release for the following 11 h to achieve a cumulative GGA release of almost 36%. Afterwards, the release of GGA from NPs was rather slow as the evidence of only 43% and 40% of drugs released from SS31-PEG-PLGA NPs and PEG-PLGA NPs in 72 h, respectively. No significant difference in released profiles was observed between both tested formulations, indicating that the modification of PLGA NPs with SS-31 did not alter the release profile of GGA.

### Gentamicin uptake

**Figure 3.**
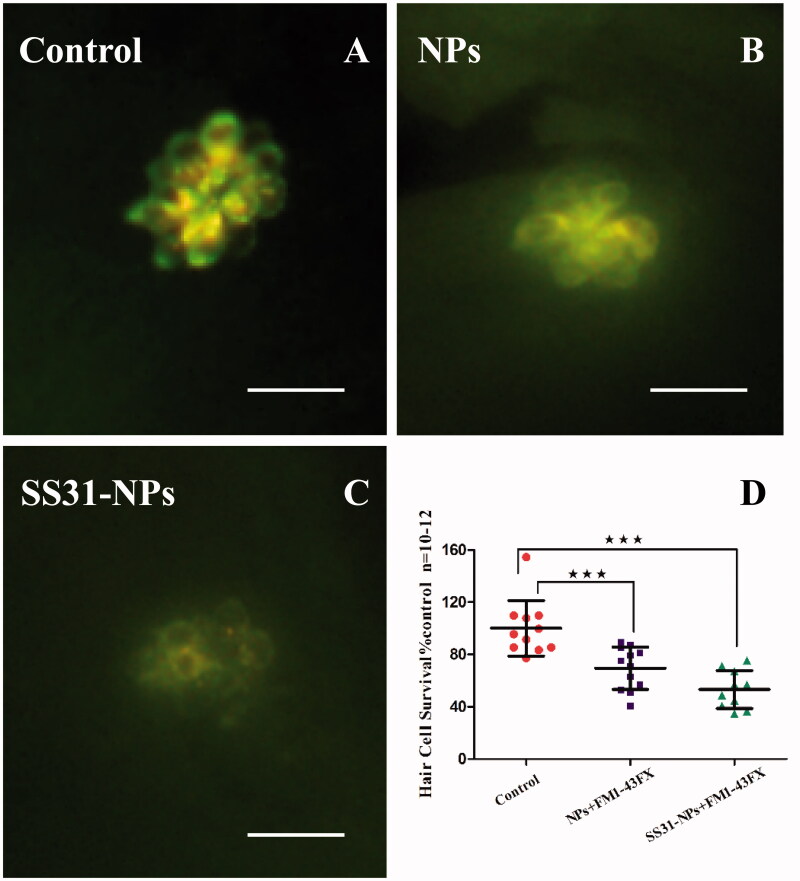
Both SS-31 modified and unmodified NPs reduced the number of FM1-43FX + cells. Example images from epifluorescent microscope showing neuromasts after FM1-43FX staining: 5 dpf zebrafish larvae were treated by EM only (A), GGA loaded NPs (B) or SS31-NPs (C) for 1 h followed by 1 μM FM1-43FX staining for 45 s. Scale bar: 10 μm. (D) The inhibition of FM1-43FX uptake in zebrafish hair cells triggered by GGA loaded unmodified NPs or SS31 modified NPs. The hair cells in larvae neuromast SO2, SO3, O1, MI1, and OC1 were counted as the percentage of the control (one-way ANOVA, ^★★★^*p* < .001).

**Figure 4.**
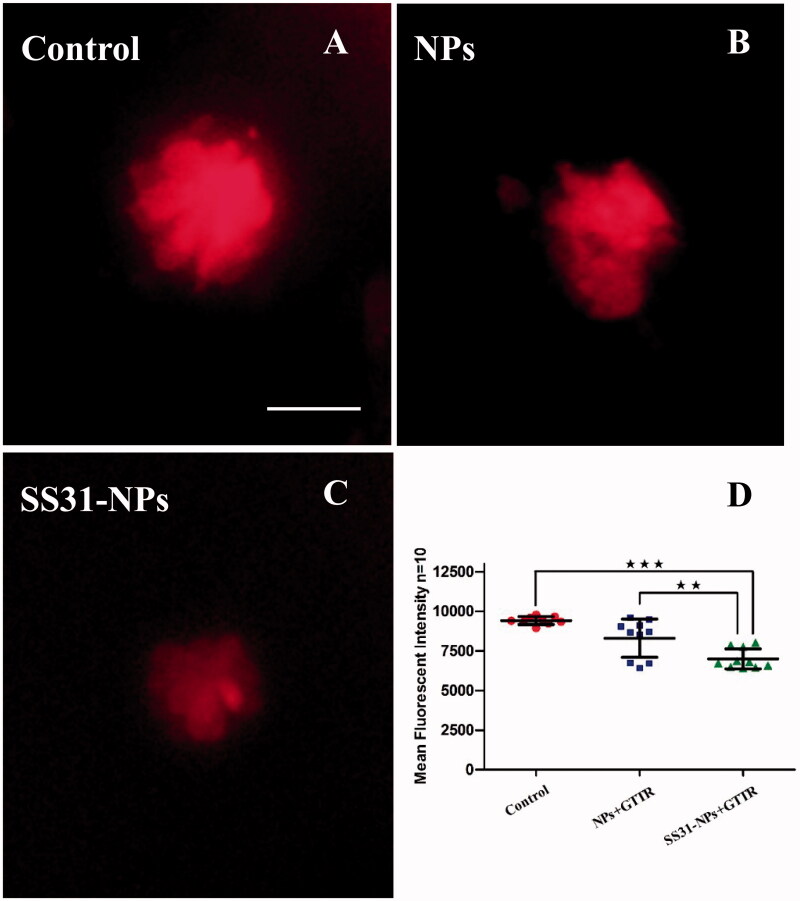
Confocal laser scanning fluorescent microscopy imaging of neuromasts in 5 dpf larvae after fluorophore Texas red tagged gentamicin (GTTR) treated for 10 min. 5 dpf zebrafish larvae were pretreated by EM only (A) GGA loaded unmodified NPs (B) or GGA loaded SS31-NPs (C) for 1 h followed by the exposure of 100 μM GTTR for 10 min. Scale bar: 10 μm. (D) SS31 modified NPs significantly reduced GTTR uptake in hair cells. The mean fluorescent intensity was quantified in five neuromasts (SO1, SO2, SO3, O1, and O2) per larvae, summed to calculate one value and averaged for each group (one-way ANOVA, ^★^*p* < .01, ^★★★^*p* < .001).

A line of studies suggest that gentamicin enters into hair cells directly through the MET channel (Alharazneh et al., 2011; Hailey et al., 2017). To investigate the effect of designed NPs on mechanotransduction (MET) channel in the zebrafish lateral line, we first evaluated the impact of them on FM1-43 uptake, which is thought to be MET-dependent processes. Pretreatment with SS31-PEG-PLGA NPs or PEG-PLGA NPs for 1 h led to a decrease in FM1-43 uptake into hair cells (Figure 3). Rapid FM1-43 uptake into hair cells was reduced to 69.5 ± 16.2% and 53.3 ± 14.5% of control fluorescence values for PEG-PLGA NPs and SS31-PEG-PLGA NPs, respectively, suggesting that MET channel was inhibited. We then examined gentamicin uptake in hair cells, addressing the relationship between loading of gentamicin and MET activity. We used gentamicin linked to the fluorescent dye TR, a rhodamine derivative, which was proved to be unable to enter hair cells alone. As seen in Figure 4, pretreatment with PEG-PLGA NPs slightly decreased gentamicin uptake, however, no significant alteration of fluorescence signal compared with the control was observed. In contrast, the uptake of gentamicin after the pretreatment of SS31-PEG-PLGA NP was dramatically reduced (74.2 ± 6.65%, *p* < .01), consistent with MET activity being critical for gentamicin loading as reported previously.

### Mitochondria targeting studies of NPs

**Figure 5.**
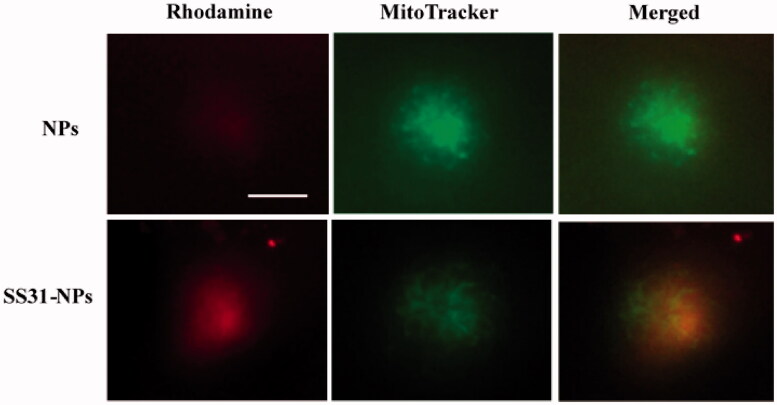
SS-31 modified NPs localization into the mitochondrial in hair cells. Zebrafish larvae (5 dpf) were pretreated with fluorescently-labeled NPs or SS31-NPs (1% of mPEG-PLGA was substituted) for 4 h and the hair cell mitochondria were labeled by 100 nM of Mitotracker Green for 30 min. Epifluorescent imaging showed mitochondrial staining by fluorescently-labeled SS-31 modified NPs. Scale bar: 10 μm.

To assess the mitochondrial targeting ability of designed NPs, the mitochondria in hair cells were labeled with the mitochondrial indicator, Mitotracker Green FM. As shown in Figure 5, densely packed mitochondria were observed in hair cells due to high metabolic load required (Owens et al., 2007). Hair cells incubated with PEG-PLGA NPs showed distributed weak fluorescent staining and no significant colocalization of NPs with MitoTracker was observed. Conversely, SS31-PEG-PLGA NP showed a much rapid internalization compared to unmodified NPs. The yellow merged image indicated that the staining of SS31-PEG-PLGA NP fitted with that of MitoTracker, providing the evidence of its mitochondrial delivery.

### Mitochondrial membrane potential

**Figure 6.**
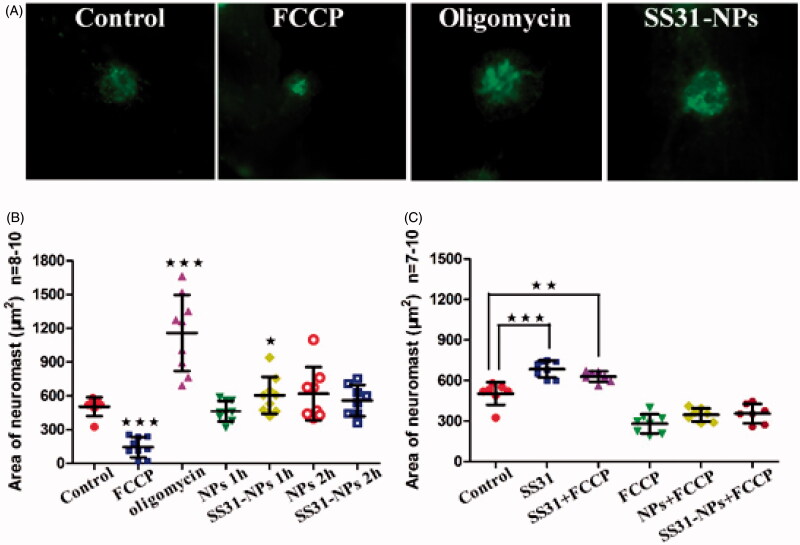
Mitochondrial membrane potential (Δ*Ψ*_m_) detection. (A) Example images from *in vivo* MMP signal. Scale bar: 10 μm. (B) Zebrafish larvae at 6 dpf were immersed EM containing diluted NPs for 1 h and 2 h or 100 μM oligomycin (positive control) for 10 min or 1 μM FCCP (negative control) for 5 min. Before evaluation, larvae were immersed in EM containing 0.1 μM DiOC_2_(3) for 30 min. After the incubation, *in vivo* imaging of zebrafish was performed using a fluorescent microscope. The area of fluorescent signal of DiOC_2_(3) at the neuromast SO1, SO2, SO3, O1, and MI1 was quantified (one-way ANOVA, ^★^*p* < .05, ^★★★^*p* < .001, compared with the control). (C) Zebrafish larvae at 6 dpf were treated by 150 μM SS31, blank NPs or blank SS-31 modified NPs for 1 h or 2 h followed by 1 μM FCCP for 5 min. Then *in vivo* imaging was conducted by fluorescent microscope and the area of five neuromasts was quantified (one-way ANOVA, ^★^*p* < .05, ^★★^*p* < .01, ^★★★^*p* < .001 compared with control).

The accumulation of SS-31 in the mitochondrial inner membrane might modulate MMP and affect mitochondrial function. Changes in the MMP after pretreatment of SS31-PEG-PLGA NPs were therefore evaluated by using DiOC_2_(3) as mitochondrial indicator. As shown in Figure 6(A), the green fluorescence area of DiOC_2_(3) significantly increased to 236.0 ± 64.6% of controls when incubated with oligomycin, which induces the mitochondrial membrane hyperpolarization. Conversely, the fluorescence signal dramatically decreased when incubated with FCCP due to the mitochondrial membrane depolarization. Both of the results indicated that *in vivo* imaging of lateral line hair cells stained with DiOC_2_(3) can be used to determine the change of MMP.

After pretreatment of SS31-PEG-PLGA NPs, the fluorescence signal initially increased by 26.2% (Figure 6(A)), however, it returned to a steady state when incubated for 2 h. On the other hand, the fluorescence signal in hair cells treated with PEG-PLGA NPs was absent to significant change as compared with the controls during the incubation (Figure 6(A)). To further explain the cause of increased MMP, FCCP was used to dissipate MMP after pretreatment of SS31 or SS31-PEG-PLGA NPs. As shown in Figure 6(B), the pretreatment with SS31 for 1 h followed by FCCP significantly increased MMP compared with treatment with FCCP alone. On the other hand, slightly increased MMP was also observed after the pretreatment of SS31-PEG-PLGA NPs. The result indicated that the reversible change of MMP observed in the group of SS31-PEG-PLGA NPs was ascribed to positive charges of SS-31 in the mitochondrial inner membranes (Sun et al., 2017).

### Dose response for gentamicin to zebrafish lateral lines hair cells

Dose responses testing for gentamicin was conducted in both acute and continuous exposures since hair cells damage induced by gentamicin is known to be distinct with treatment length.

**Figure 7.**
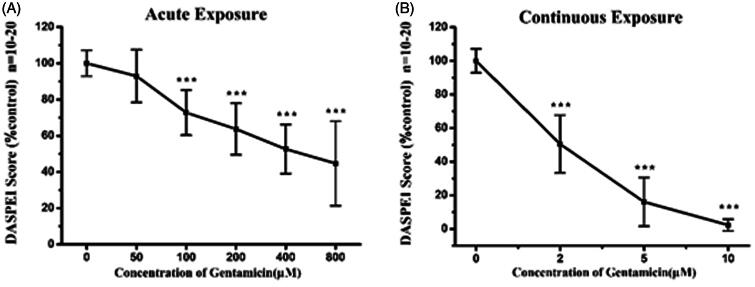
Gentamicin induced different amount of damage dependent on the time of exposure. Loss of DASPEI staining in neuromasts as a function gentamicin concentration is shown as compared to the mocktreated controls. The 5 dpf zebrafish larvae was immersed in EM containing a series concentrations of gentamicin for 1 h (acute exposure) (A) or for 6 h (continuous exposure) (B) respectively then stained by 0.005% DASPEI for 20 min. Recovery time (1 h) before DASPEI staining was needed in acute exposure while was not necessary in continuous exposure (one-way ANOVA, ^★★★^*p* < .001 compared with control).

Figure 7 illustrates that the dose–response relationship by analyzing DASPEI labeling of lateral lines neuromasts after acute or chronic exposure to gentamicin ranging from 0 to 800 μM. As shown in Figure 7(A), DASPEI scores decreased to 63.7 ± 14.2% of the control group following 200 μM gentamicin treatments, which reflected significant hair cell loss after acute exposure. Gradually increased hair cell damage was observed when incubated with higher concentrated gentamicin upon acute exposure. On the contrary, continuous gentamicin exposure induced substantial hair cells loss as compared with that observed in the acute exposure. Figure 7(B) shows that the DASPEI scores was 50.5 ± 17.2% of the control following only 2 μM gentamicin continuous exposure, while it quickly decreased to 16.0 ± 14.5% following 5 μM gentamicin and nearly 0% following 10 μM gentamicin.

### SS31-PEG-PLGA NPs protect zebrafish lateral hair cells against gentamicin

**Figure 8.**
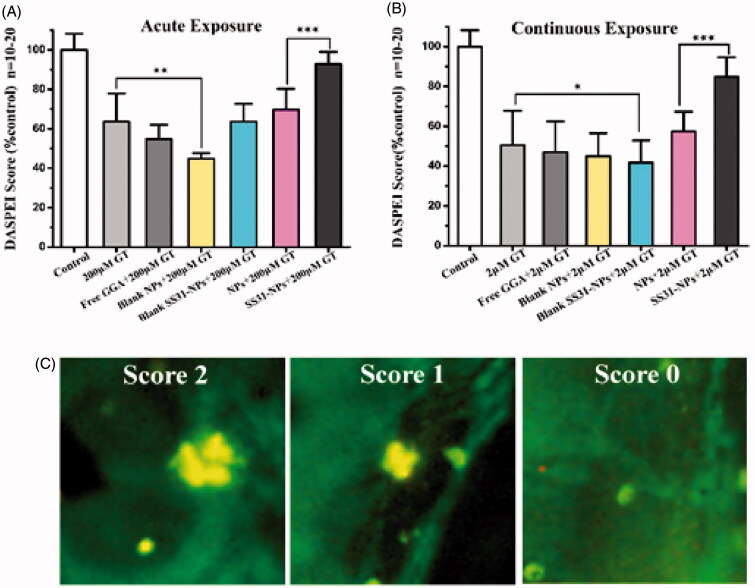
SS-31 modified NPs protect hair cells from gentamicin in both of acute and chronic exposure. The survival of hair cells was evaluated by DASPEI staining. (A) SS-31 modified NPs conferred complete protection from 200 μM gentamicin in the acute exposure. (B) SS-31 modified NPs conferred nearly complete protection from 2 μM gentamicin in the chronic exposure (one-way ANOVA, ^★^*p* < .05, ^★★^*p* < .01, ^★★★^*p* < .001). (C) Example images for DASPEI staining for evaluating the 10 neuromasts by scores (0 for no/little staining, 1 for reduced staining, 2 for full staining). Scale bar: 10 μm.

To evaluate the response of hair cells to GGA loaded NPs against gentamicin, pretreatment with designed formulations for 1 h followed by 1 h or 6 h of co-treatment with gentamicin was performed. Groups of zebrafish were then evaluated for each condition and their DASPEI score was then compared to that of mock-treated control. For acute exposure, SS31-PEG-PLGA NPs protected hair cells against gentamicin as the evidence of increased hair cell survival from 63.7 ± 14.2% (gentamicin only) to 92.8 ± 6.14% (Figure 8(A); *p* < .001). In contrast, neither free GGA nor GGA loaded PLGA-PEG NPs larvae provided protection from gentamicin. Furthermore, the pretreatment with SS31-PEG-PLGA NPs followed by co-treatment with gentamicin for 6 h significantly increased hair cell survival from 50.5 ± 17.2% (gentamicin only) to 84.9 ± 9.6% (Figure 8(B); *p* < .001) while the other groups failed to protect lateral lines hair cells against gentamicin.

**Figure 9.**
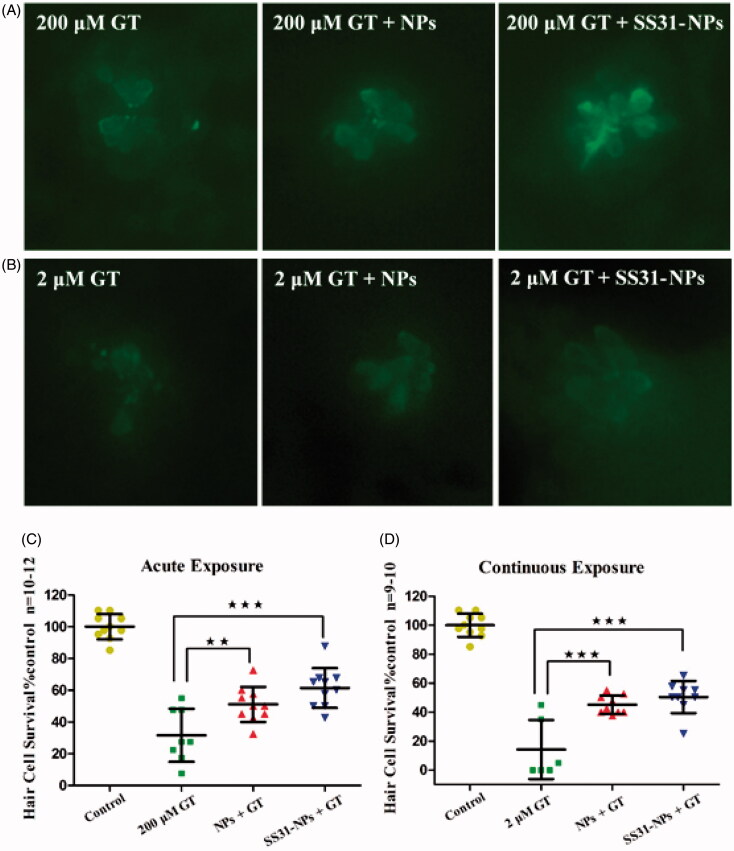
Quantitative evaluation for hair cell survival using immunocytochemistry. *In vivo* imaging of hair cells in zebrafish lateral line upon acute exposure (A) and continuous exposure (B) of gentamicin. Scale bar: 10 μm. The hair cell number was quantified in five neuromasts (O1, O2, MI1, M2, and OP1) per larvae, summed to calculate one value per animal, and averaged for each group. Results were displayed as the mean hair cell survival as a percentage of the group treated only in EM. The 5 dpf zebrafish larvae groups were treated with 200 μM gentamicin (GT) for 1 h, GGA loaded NPs and SS31-NPs for 1 h respectively then followed by 200 μM GT for 1 h (C) or 2 μM GT for 6 h (D) (one-way ANOVA, ^★★^*p* < .01, ^★★★^*p* < .001).

To confirm DASPEI scores, direct hair cells count by immunohistochemically anti-parvalbumin stain to visualize hair cells was conducted after the pretreatment of NPs. The data further supported that SS31-PEG-PLGA NPs robustly protected hair cells from gentamicin damage in either acute or continuous exposure. Pretreatment for 1 h with SS31-PEG-PLGA NPs followed by acute exposure of gentamicin significantly increased hair cell survival from 31.6 ± 16.7% by gentamicin alone to 61.4 ± 12.6% (Figure 9; *p* < .001). Similarly, the survival rate of hair cells upon the pretreatment of SS31-PEG-PLGA NPs significantly improved upon the continuous exposure of gentamicin (from 14.2% to 50.4%). On the other hand, pretreatment for 1 h with GGA loaded PLGA-PEG NPs followed by co-treatment with gentamicin also resulted in slightly increased hair cell survival despite of lower significant difference observed in either acute or chronic exposure.

## Discussion

The present study demonstrated that a novel mitochondrial targeting delivery system protects lateral line hair cells from both acute and chronic gentamicin ototoxicity. Zebrafish lateral line hair cells were used as model in this study because of their high similarity to mammalian inner ear hair cells in structures and functions (Esterberg et al., 2013). Other advantages including high sensitivity and easy to scale up make it very favorable to be an evaluating system for screening ototoxic agents or protectants (MacRae & Peterson, 2015). Previous studies have revealed that targeting mitochondria provided superior protective efficacy against aminoglycoside exposure when compared with untargeted therapeutics molecules or cargo. Our findings indicated that mitochondrial targeted therapies driving by SS-31 have therapeutic potential to ameliorate toxic effects of aminoglycoside exposure.

In general, the uptake of gentamicin played a key role on its toxic effects. Numerous studies suggest requirement for MET activity for aminoglycosides loading, and thus the known MET channel blockers, such as amiloride, inhibited their loading by reducing MET activity (Owens et al., 2009; Hailey et al., 2017). Consistent with the previously reports, our results demonstrated that SS31-PEG-PLGA NPs reduced both MET activity and gentamicin entry, which seem to partially explain their protective effect. Recently, Dale et al. revealed that aminoglycosides enter into hair cells via both endocytic and non-endocytic pathways (Hailey et al., 2017). It was inferred that aminoglycosides could be directly delivered to cytosol via MET channel, regardless of endocytic pathway. The cytosolic fraction of gentamicin induced hair cells damage immediately (in ‘acute response’ pattern) and thus the blockage of MET channel appeared to prevent acute toxicity associated with gentamicin. Our results showed that both SS31-PEG-PLGA NPs and PEG-PLGA NPs reduced the activity of MET channel, however, only SS31-PEG-PLGA NPs significantly decreased ∼25% gentamicin loading as compared with control. It indicated that alternative entry mechanisms presented in addition to MET channel. The endocytic processes was recognized to be involved, by which gentamicin was transferred to lysosomes of hair cells. The lysosomal delivery allowed transient isolation of gentamicin from toxic targets, such as mitochondria. Nevertheless, it demonstrated that the possible discrepancy of intracellular distribution led to no statistical protective efficiency against gentamicin after the pretreatment of PEG-PLGA NPs (Figure 8).

To monitor mitochondrial dysfunction upon gentamicin, we initially used a mitochondrial potentiometric dye, DASPEI, to image hair cells (Coffin et al., 2013). Figure 8 demonstrates that SS31-PEG-PLGA NPs provided superior protection against gentamicin in either acute or chronic exposure when compared with PEG-PLGA NPs. Since gentamicin loading into hair cells was reduced but not blocked after the pretreatment of SS31-PEG-PLGA, protection act was complicated. The modulation of intracellular signaling pathway cannot be excluded from the attenuation of gentamicin-induced hair cells. To clarify the protective mechanism of SS31-PEG-PLGA NPs, we then applied the blank formulations to pretreat zebrafish. The fact that blank SS31-PEG-PLGA reduced MET activity and gentamicin loading was unable to execute any mitochondrial protection against ototoxic molecules (as shown in Figure 8). These data indicated that the majority of protective effect in SS31-PEG-PLGA NPs was attributed to the intracellular delivery of GGA, a 70 kDa heat shock protein (HSP 70) inducer. The direct delivery of GGA to mitochondria by SS31-PEG-PLGA (as shown in Figure 6), where HSP chaperones associated with cyclophilin D (CypD), eventually preserved mitochondrial integrity and shut off the initiation of cell death.

Numerous studies revealed that mitochondrial swelling was the earliest toxic and most prevalent event alongside the loss of MMP in aminoglycosides-induced hearing loss (Dehne et al., 2002; Owens et al., 2007). Moreover, the important mitochondrial functions, such as regulation of the cell redox state, transport of metabolites, lipid and amino acid metabolism and cell death, are highly dependent on MMP (Solaini et al., 2007). Therefore, MMP was frequently used to predict hair cell survival after the treatment of protectant or ototoxic molecules. To further investigate the impact of formulation, MMP was measured using DiOC_2_(3) as a potential indicator. Considering the transient increased MMP observed after the pretreatment of SS31-PEG-PLGA, we then investigated the source of protons in mitochondria using FCCP, which dissipated MMP via transport of protons across the inner mitochondrial membrane. As shown in Figure 5, improved MMP was observed in the pretreatment of SS31 or SS31-PEG-PLGA when compared with treatment with FCCP alone, indicating that formed increased MMP was due to positively charged SS31. However, further studies that investigate the effect of SS31-PEG-PLGA induced changes of MMP on mitochondrial ATP production, coupling of oxidative phosphorylation and cell energy state were out of the scope of the present study since the absence of corresponding approaches to harvest hair cells from zebrafish.

The designed SS31-PEG-PLGA NPs was expected to be locally administrated via the round window membrane to the inner ear, which provides an alternative noninvasive technology to bypass the blood–cochlear barriers (Salt & Plontke, 2009). Intratympanic dexamethasone is now being evaluated in phase IV clinical trial for the treatment of cisplatin-induced ototoxicity (https://clinicaltrials.gov). To mimic the circumstance after intratympanic injection, a transwell plate was used to predict *in vitro* release of NPs. Since round window membrane was permeable to small NPs, transwell plates with the pore size of 400 nm were selected to monitor drug release. In addition, HEPES was also used to mimic the perilymph in inner ear due to relatively low protein in the inner ear fluids.

## Conclusions

In summary, we demonstrated that SS-31 modified PLGA NPs improved the protection efficiency of GGA against gentamicin-induced hair cell damage in zebrafish. Although decreased gentamicin loading in hair cells after the pretreatment of SS31-PEG-PLGA NPs, the pharmacological effect of the designed formulation was mainly attributed to their mitochondrial delivery profile, which was capable to regulate the homeostasis within mitochondria against ototoxic agents. Thus, the functionalizing nanoparticles with SS-31 might be ideally suited for preventing hearing loss in humans. The further testing in mammals needs to investigate the protective effects in the inner ear as well.

## Supplementary Material

Hongzhuo_Liu_et_al_supplemental_content.zip
