# Supplementary material for: SS-31 peptide enables mitochondrial targeting drug delivery: a promising therapeutic alteration to prevent hair cell damage from aminoglycosides
Source: Drug Deliv. 2017 Dec 7;24(1):1750–61. doi: 10.1080/10717544.2017.1402220 (PMC8241023; doi:10.1080/10717544.2017.1402220)
Supplement: Hongzhuo_Liu_et_al_supplemental_content.zip [file IDRD_A_1402220_SM9404.zip › Hongzhuo Liu et al supplemental content.docx]

Supporting information

**SS-31 peptide enables mitochondrial targeting drug delivery: a promising therapeutic alteration to prevent hair cell damage from aminoglycosides**

Xiao Kuang, Shuang Zhou, Weiling,Guo, Zhenjie Wang, Yanhui Sun, Hongzhuo Liu^*^

School of Pharmacy, Shenyang Pharmaceutical University, Shenyang 110016, China

* Corresponding author.

Hongzhuo Liu Mail address: School of Pharmacy, Shenyang Pharmaceutical University, No. 103 Wenhua Road, Shenyang 110016, P.R. China; Tel.: +8624 43520586; Email address: liuhongzhuo@syphu.edu.cn


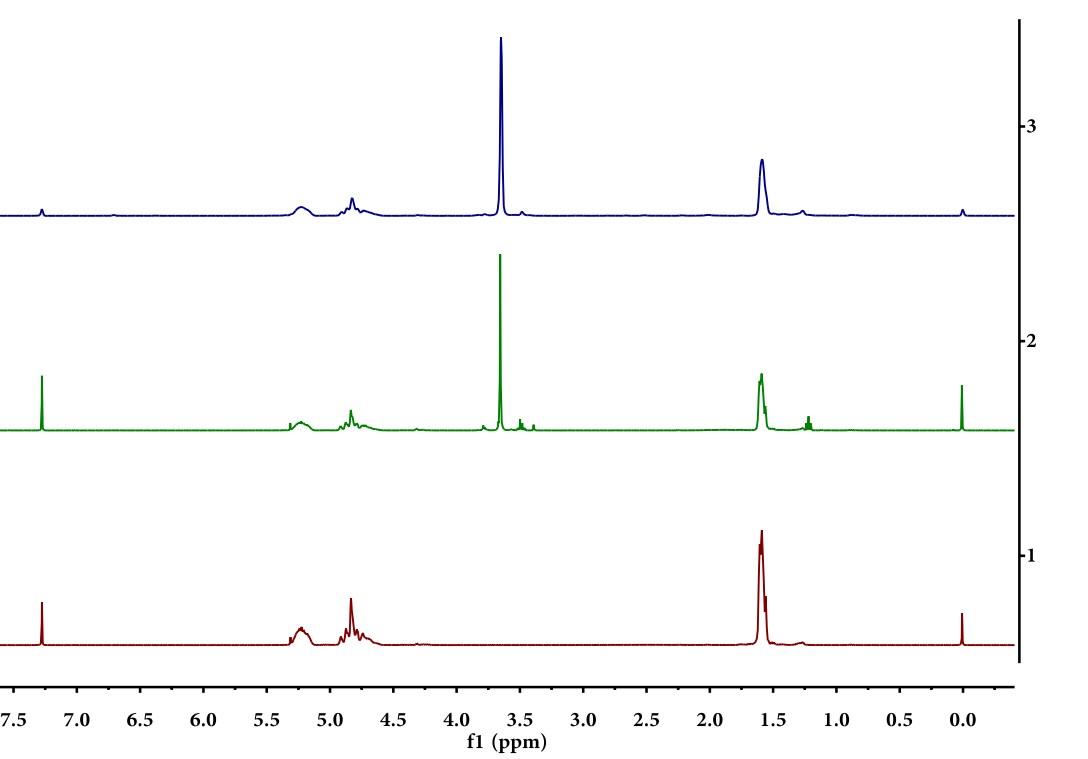


**A**


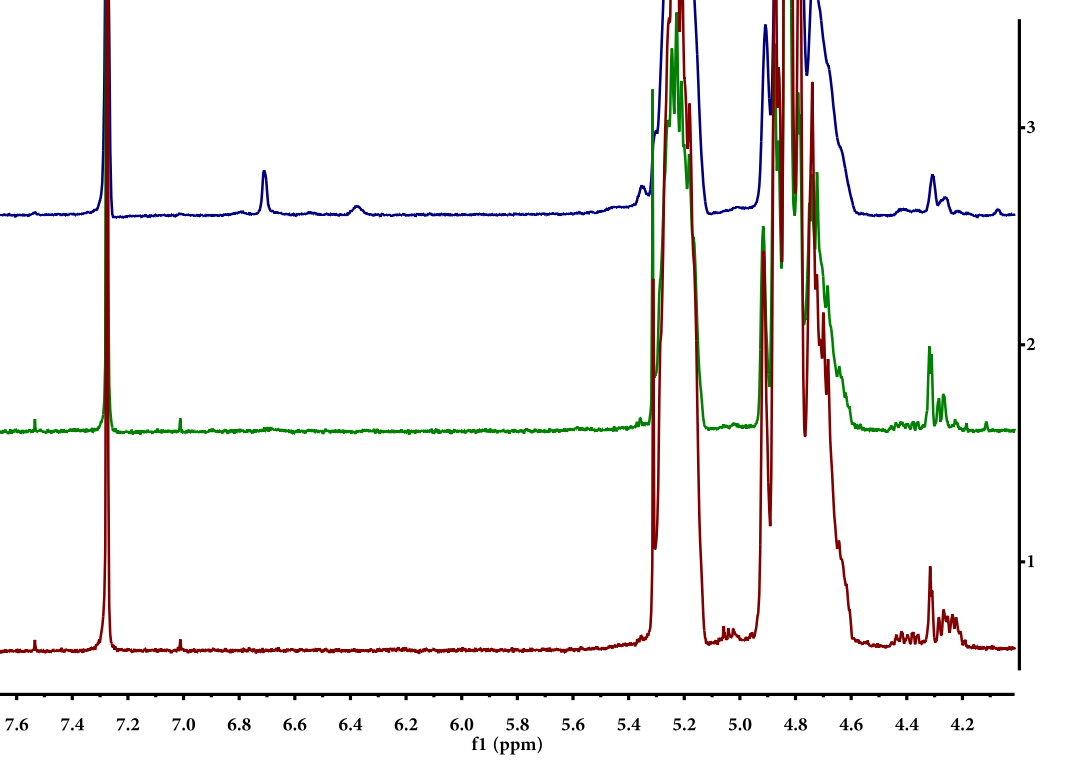


**B**

**Fig.S1.** (**A**) The ^1^H-NMR (CDCl_3_) spectra of PLGA (1), PLGA-b-mPEG (2) and PLGA-b-PEG-mal (3), δ7.3 [CDCl_3_], 5.2 [(OCHCH_3_C(O)], 4.8 [(OCH_2_C(O))], 3.6 [(OCH_2_)], 1.6 [(CH_3_CH)], δ6.7[CH=CH] in maleimide. (**B**) Proportionally stretched ^1^H-NMR (CDCl_3_) spectra of **A**, PLGA (1), PLGA-b-mPEG (2) and PLGA-b-PEG-mal (3).

**Table S1.** The molar ratio (%) of elements on the surface of nanoparticles by XPS

| Sample | C | O | N | S |
| --- | --- | --- | --- | --- |
| NPS | 67.77 | 30.34 | 1.75 | 0.14 |
| Drug loaded NPS | 65.08 | 33.01 | 1.76 | 0.16 |
| Targeted Drug loaded NPS | 62.23 | 35.5 | 2.01 | 0.26 |

**Fig.S2.** Binding energy of elements on the surface of NPs (A), GGA loaded NPs (B) and GGA loaded SS31-PEG-PLGA NPs (C).

**B**

**A**

**C**

**Modification of SS31 onto the surface of NPs**

SS31-Cys (0.267 mg) was added into 1 ml of maleimide-PEG-PLGA NPs (10 mg/ml) to ensure peptide: maleimide–PEG–PLGA NPs mass ratio of 1.3:1 in PBS 7.4. After reacting in a shaking bath at low speed for 1 h, the mixture were concentrated by centrifuging (13000 g, 30 min) at 4 ℃ and the supernatant collected was mildly reduced by TECP (tris(2-carboxyethyl)phosphine hydrochloride, Meilunbio, Dalian, China) to generate free sulfhydryls, followed by HPLC determination to confirm the conjugation efficacy of SS31. The analysis was performed using a Hitachi HPLC system (Tokyo, Japan) equipped with a RP-HPLC column (Inertsil ODS-SP C_18_ column, 5 μm, 4.6 mm×250 mm). HPLC parameters were as follows: flow rate, 1.0 ml/min; mobile phase A was 0.1% trifluoroacetic in acetonitrile, and mobile phase B was 0.1% trifluoroacetic in water; wavelength and column temperature were set at 220 nm and 25 ℃ respectively. The gradient program was 0-25 min 15-40% A, 25-30 min 100% A. The injection volume was 10 μl of each sample.

**Synthesis of gentamicin-Texas red conjugate**

Gentamicin sulfate (GT) and Texas Red (TR) succinimidyl ester were agitated together to produce gentamicin tagged with fluorophore Texas red (GTTR). Briefly, 4.4 ml of 50 mg/ml GT was mixed with 0.6 ml of 2 mg/ml Texas Red succinimidyl esters with gentle agitating for 8 days at 4 ℃. The molar ratio of GT: GTTR for resulted product was about 300:1 after reaction. HPLC method was used for the determination of GTTR. Chromatography separation was achieved using an Inertsil ODS-SP column (4.6 mm×150 mm, 5 μm) at RT. Gradient elution mode was applied at the flow rate of 1 ml/min with the mobile phase of acetonitrile (A)-water (B). The gradient program was 0-2 min 5% A, 2-18 min 5-70% A and 18-20 min 70% A. The detection wavelength was set at 595 nm. The injection volume was 10 μl of each sample.
